# Pneumovesicoscopic *en bloc* resection of multifocal bladder paraganglioma: a case report

**DOI:** 10.3389/fmed.2026.1992514

**Published:** 2026-10-21

**Authors:** Bing Wang, Yu Hong, Tianhai Liao, Dingli Hu, Yunlong Li

**Affiliations:** Department of Urology, First People's Hospital of Kunshan, Kunshan, China

**Keywords:** *en bloc* resection, minimally invasive surgery, paraganglioma, pneumovesicum, urinary bladder

## Abstract

**Background:**

Bladder paraganglioma is a rare non-epithelial neuroendocrine tumor. Catecholamine release may cause micturition-related headache, palpitations, sweating, paroxysmal hypertension, or syncope, but cases without typical symptoms can be difficult to recognize before surgery. Complete surgical excision remains the mainstay of treatment.

**Case presentation:**

A 75-year-old woman was admitted after two bladder lesions were detected. She had no hematuria, lower urinary tract symptoms, or micturition-related headache, palpitations, sweating, or paroxysmal hypertension. Contrast-enhanced CT showed two hypervascular nodules near the bladder neck and on the left posterior wall, measuring 12 × 14 mm and 6 × 9 mm, respectively. Twenty-four-hour urinary free catecholamines were within the reference ranges. Plasma free normetanephrine was mildly elevated at 0.96 nmol/L (upper reference limit, 0.71 nmol/L; approximately 1.35 × ULN) when measured by LC-MS/MS under standardized sampling conditions, whereas plasma metanephrine and 3-methoxytyramine remained within their reference ranges. This profile was considered consistent with a clinically silent but mildly biochemically functional tumor. The patient underwent pneumovesicoscopic *en bloc* resection. Histopathology confirmed multifocal bladder paraganglioma confined to the submucosa, with negative surgical margins, a Ki-67 proliferation index <1%, and retained SDHB expression. Recovery was uneventful, and cystoscopy showed no recurrence at 12 months.

**Conclusions:**

Bladder paraganglioma should be considered in a hypervascular submucosal bladder mass with intact overlying mucosa even when typical catecholamine-related symptoms are absent. This single case supports the technical feasibility of pneumovesicoscopic *en bloc* resection for selected localized lesions, but the 12-month follow-up is insufficient to establish comparative effectiveness or long-term oncologic safety.

## Background

Bladder paraganglioma is a rare non-epithelial neuroendocrine tumor of the urinary bladder, accounting for approximately 0.05%–0.06% of bladder tumors and only a small proportion of pheochromocytomas and paragangliomas (PPGLs) (1–4). It is thought to arise from sympathetic paraganglionic tissue within the bladder wall and may involve the submucosa or muscular layer. These tumors can synthesize and secrete catecholamines (1, 4, 5). Some patients develop paroxysmal hypertension, headache, palpitations, sweating, or syncope during bladder filling, micturition, or shortly afterward, whereas others have no typical secretory symptoms and present with painless hematuria or an incidentally detected bladder mass (1–3, 6). The absence of typical symptoms therefore does not exclude biochemical catecholamine activity.

Imaging cannot establish the diagnosis of bladder paraganglioma on its own, but it can provide useful preoperative clues. Lesions are typically round or oval, relatively well circumscribed, and often grow toward the bladder lumen with an intact mucosal surface. Marked enhancement is common on contrast-enhanced CT, and some lesions remain strongly enhanced in the venous or delayed phase. MRI may show mildly high T2 signal, restricted diffusion, and a rapid-enhancement/slow-washout pattern (7–12). These features differ from the more irregular mucosa-based appearance of many urothelial tumors, although overlap exists with other submucosal lesions such as leiomyoma and hemangioma. Histopathology and immunohistochemistry remain necessary for definitive diagnosis.

We report a case of multifocal bladder paraganglioma in a patient without typical catecholamine-related symptoms but with mild biochemical catecholamine activity. The tumors were treated by pneumovesicoscopic *en bloc* resection. The imaging, biochemical findings, perioperative management, pathology, and follow-up are discussed with particular attention to the practical features of this approach.

## Case presentation

A 75-year-old woman was admitted on November 30, 2024, after bladder lesions had been detected for more than 1 week. She had no hematuria, urinary frequency, urgency, dysuria, abdominal pain, or typical catecholamine-related symptoms such as micturition-associated headache, palpitations, sweating, syncope, or paroxysmal blood-pressure elevation. She had a > 20-year history of hypertension treated with amlodipine besylate, with stable blood-pressure control, and a > 20-year history of diabetes mellitus treated with acarbose and repaglinide. She had undergone radical surgery for right breast cancer 5 years earlier and cholecystectomy 11 years earlier. She denied a family history of pheochromocytoma, paraganglioma, or a known hereditary tumor syndrome in first- or second-degree relatives.

On admission, her temperature was 36.0 °C, heart rate 86 beats/min, respiratory rate 15 breaths/min, and blood pressure 116/83 mmHg. Cardiopulmonary and abdominal examinations were unremarkable. There was no costovertebral-angle tenderness and no suprapubic tenderness or palpable mass.

Contrast-enhanced CT showed two well-defined nodules near the bladder neck and on the left posterior wall, measuring 12 × 14 mm and 6 × 9 mm, respectively. Both lesions were hypervascular. The larger lesion increased from 30 HU on unenhanced imaging to 113 HU in the arterial phase and 204 HU in the venous phase; the smaller lesion also showed clear venous-phase enhancement (Table 1). Both adrenal glands were normal in morphology and attenuation, and no definite metastatic lesion was seen within the scanned field. A bladder neoplasm was initially considered, while ectopic pheochromocytoma/bladder paraganglioma could not be excluded. Because contrast-enhanced CT together with cystoscopy provided adequate information on lesion size, location, and the relevant anatomy for surgical planning, MRI was not obtained preoperatively. Cystoscopy showed two round or hemispheric submucosal protrusions, approximately 1.5 cm and 0.8 cm in maximum diameter, with smooth, intact mucosa and visible surface vessels. Correlating the endoscopic and CT findings, the larger lesion was near the bladder neck and adjacent to the trigone, while the second was on the left posterior wall near the trigone. Both ureteral orifices were clearly identified without obvious involvement (Figure 1). Prophylactic ureteral stents were not placed because the orifices were clearly visualized and neither lesion involved the ureteral openings.

**Table 1:** Contrast-enhanced CT attenuation values of the two bladder lesions.

Location	Size/mm	Unenhanced/HU	Arterial/HU	Venous/HU	Excretory/HU	Maximum enhancement/HU
Near bladder neck	12 × 14	30	113	204	172	174
Left posterior wall	6 × 9	45	57	133	109	88

**Figure 1:**
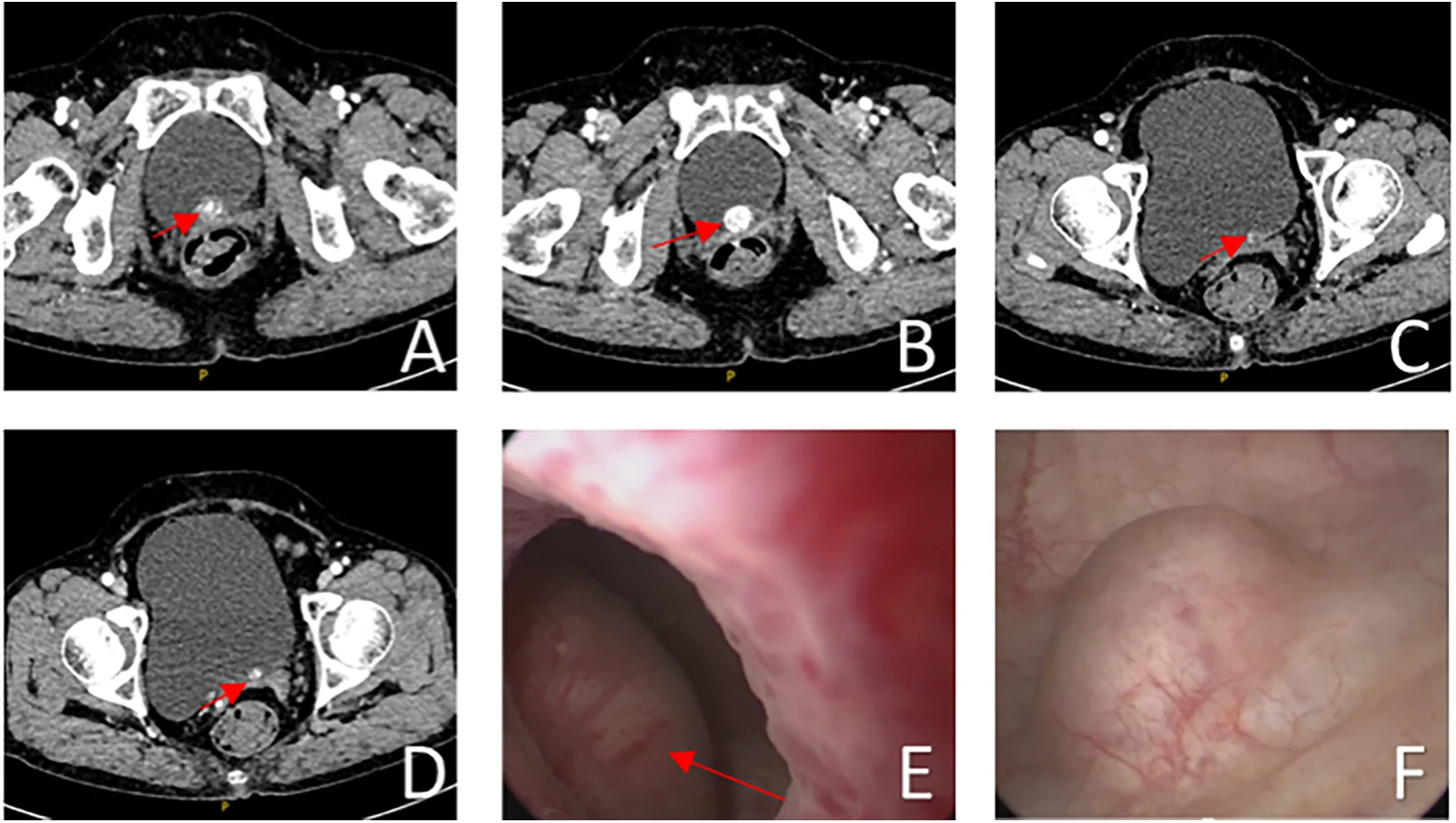
Preoperative contrast-enhanced CT and cystoscopic findings. **(A,B)** Arterial- and venous-phase CT images of the lesion near the bladder neck; **(C,D)** arterial- and venous-phase CT images of the left posterior-wall lesion; red arrows indicate the lesions. **(E,F)** Cystoscopic views of two round/hemispheric submucosal protrusions with smooth, intact overlying mucosa.

Biochemical testing showed 24-h urinary free epinephrine, norepinephrine, and dopamine within their respective reference ranges. Plasma free normetanephrine (NMN) was mildly elevated at 0.96 nmol/L (upper reference limit, 0.71 nmol/L; approximately 1.35 × ULN), whereas plasma free metanephrine (MN) and 3-methoxytyramine (3-MT) were normal; vanillylmandelic acid (VMA) and homovanillic acid (HVA) were mildly elevated (Table 2). Blood for plasma MN, NMN, and 3-MT was collected after fasting and 20–30 min of quiet supine rest and analyzed by liquid chromatography-tandem mass spectrometry (LC-MS/MS). The mildly elevated NMN result was not repeated before surgery. Her long-term medications were amlodipine, acarbose, and repaglinide.

**Table 2:** Preoperative biochemical findings. Plasma NMN, MN, and 3-MT were measured by LC-MS/MS under standardized sampling conditions.

Category	Specimen	Analyte	Result	Reference range	Status
Catecholamines	24-h urine	Free epinephrine	1.6 μg/24 h	0–20 μg/24 h	Normal
Catecholamines	24-h urine	Free norepinephrine	5.7 μg/24 h	0–90 μg/24 h	Normal
Catecholamines	24-h urine	Free dopamine	77.6 μg/24 h	0–600 μg/24 h	Normal
Metanephrines	Plasma	Free normetanephrine (NMN)	0.96 nmol/L	≤0.71 nmol/L	Elevated
Metanephrines	Plasma	Free metanephrine (MN)	0.15 nmol/L	≤0.42 nmol/L	Normal
Metanephrines	Plasma	3-Methoxytyramine (3-MT)	0.04 nmol/L	≤0.26 nmol/L	Normal
Other metabolites	Plasma	Vanillylmandelic acid (VMA)	106.00 nmol/L	12–62 nmol/L	Elevated
Other metabolites	Plasma	Homovanillic acid (HVA)	203.00 nmol/L	14.2–160 nmol/L	Elevated
Catecholamines	Plasma	Dopamine	0.19 nmol/L	≤0.31 nmol/L	Normal
Catecholamines	Plasma	Norepinephrine	0.79 nmol/L	0.6–5.3 nmol/L	Normal
Catecholamines	Plasma	Epinephrine	0.09 nmol/L	≤0.35 nmol/L	Normal

Although the patient had no typical catecholamine-related symptoms, the mild elevation in plasma NMN suggested biochemical catecholamine activity. Taken together with normal 24-h urinary catecholamines, MN, and 3-MT, the tumor was considered clinically silent but mildly biochemically functional. Because NMN was only about 1.35 × ULN and was not repeated preoperatively, this isolated elevation was interpreted cautiously and was not considered evidence of marked catecholamine excess.

An endocrinology consultation was obtained before surgery. Given the absence of typical secretory symptoms and the modest biochemical abnormality, no preoperative alpha-adrenergic blockade or PPGL-specific volume expansion was used. Perioperative management instead emphasized close blood-pressure and heart-rate monitoring, with the anesthesia team prepared to treat hemodynamic changes as they occurred.

The operation was performed under endotracheal general anesthesia. A radial arterial catheter was placed for continuous invasive blood-pressure monitoring, with continuous monitoring of heart rate, SpO₂, and EtCO₂. During anesthesia, systolic blood pressure ranged from approximately 117 to 170 mmHg and diastolic pressure from 45 to 68 mmHg; heart rate was mostly 52–80 beats/min, with a brief increase during emergence. SpO₂ remained 98%–100% and EtCO₂ 27–40 mmHg. Esmolol 20 mg, ephedrine 6 mg, and atropine 0.5 mg were administered according to hemodynamic changes. No sustained severe hypertension, severe hypotension, or hemodynamic event requiring termination of the procedure occurred. In the lithotomy position, a 22-Fr cystoscope was inserted. Under direct vision, a 10-mm trocar was placed through the lower midline abdominal wall and a 5-mm trocar approximately 3 cm medial to the left anterior superior iliac spine. The Foley catheter balloon was inflated to occlude the bladder outlet and minimize CO₂ leakage. A STORZ constant-pressure CO₂ insufflation system was used to establish pneumovesicum at 11 mmHg, with pressure maintained by the system's feedback control. Pneumovesicum was maintained for 106 min.

The planned resection margin was marked with electrocautery approximately 5 mm from the visible tumor edge. An ultrasonic energy device was used to incise the bladder wall along the marking and to continue dissection along the tumor boundary. Both lesions were removed *en bloc* and appeared grossly completely excised intraoperatively, then placed in a specimen retrieval bag and extracted (Figure 2). Total operative time was 180 min and estimated blood loss was approximately 10 mL. The bladder wall was closed continuously with a 3-0 absorbable suture. After closure, the bladder was filled with normal saline through the catheter; no leakage was seen along the suture line. A 22-Fr three-way Foley catheter was left in place to maintain low-pressure bladder drainage and reduce early tension on the repair.

**Figure 2:**
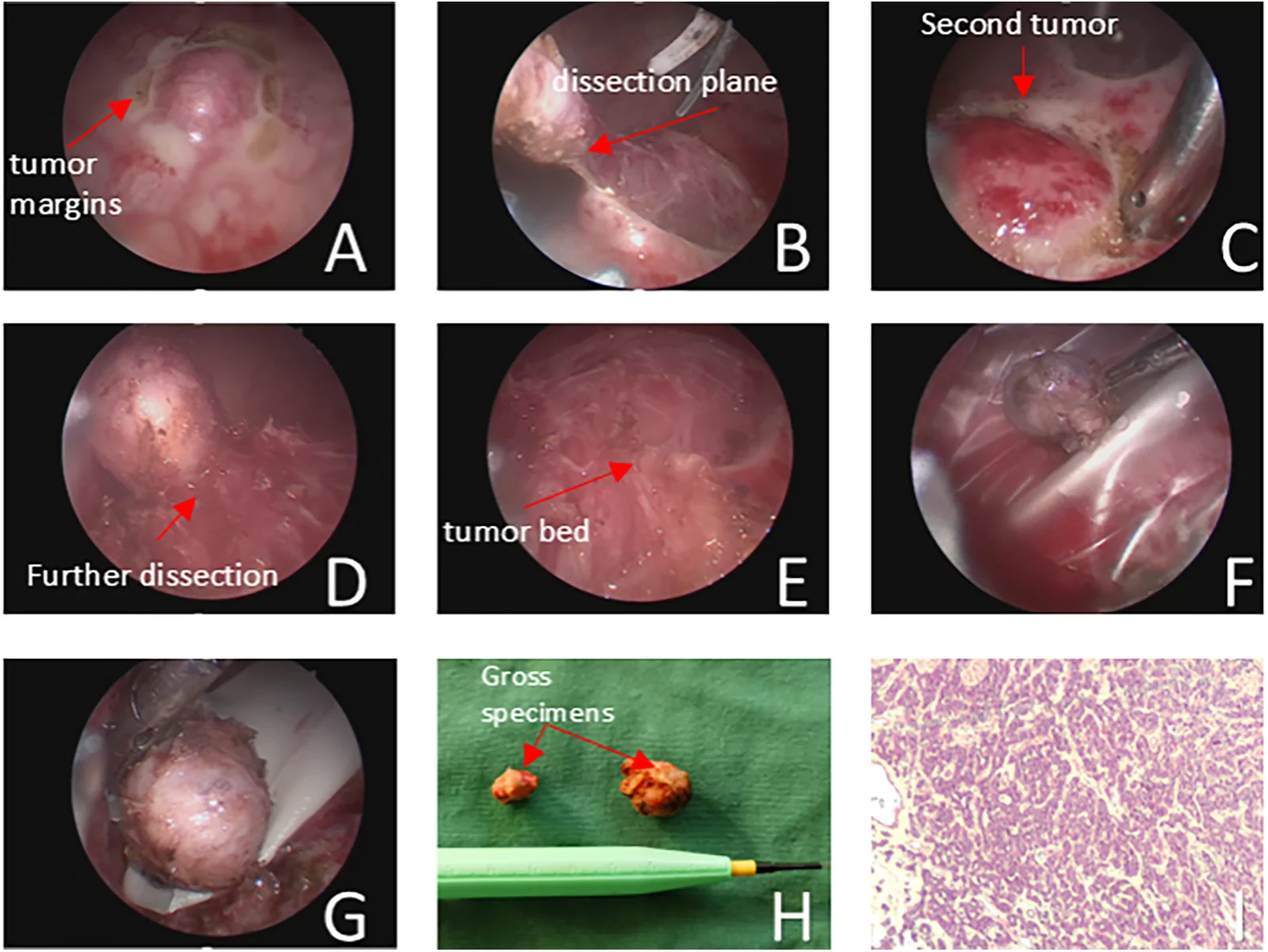
Pneumovesicoscopic *en bloc* resection and pathology. **(A)** Tumor boundary marking; **(B)** initial dissection plane; **(C)** second tumor; **(D)** further dissection along the tumor plane; **(E)** tumor bed; **(F,G)** intact tumors in the retrieval bag; **(H)** gross specimens; **(I)** H&E staining showing nested/organoid Zellballen architecture (×200).

Gross examination showed two specimens measuring 1.0 × 0.8 × 0.5 cm and 2.0 × 1.5 × 1.0 cm; both were gray-white and solid on cut surface. Histologically, the tumor cells were arranged in characteristic nested (Zellballen) and organoid patterns separated by delicate fibrovascular septa. The tumors were confined to the bladder submucosa. Mitotic figures were rare, with no tumor necrosis, lymphovascular invasion, or perineural invasion. Surgical margins were free of tumor. Immunohistochemistry showed positivity for Syn, CgA, CD56, NSE, GATA3, and S-100, negativity for AE1/AE3, a Ki-67 proliferation index <1%, and retained SDHB expression, supporting the diagnosis of bladder paraganglioma.

Continuous bladder irrigation was maintained for 12 h. Before catheter removal, a routine CT scan, rather than retrograde cystography or CT cystography, showed no obvious perivesical fluid collection. In the absence of abdominal pain, fever, or other clinical findings suggestive of urinary leakage, the Foley catheter was removed on postoperative day 7, after which the patient voided normally. The postoperative hospital stay was 7 days. No urinary leakage, urinary tract infection, clinically significant hematuria, urinary retention, or ureteral obstruction occurred. Recorded postoperative blood pressure was approximately 116–133/58–83 mmHg, with no evident cardiovascular event. NMN and other metanephrines were not rechecked after surgery. At 12 months, the patient had no voiding discomfort and cystoscopy showed no tumor recurrence.

## Discussion

The clinical phenotype of bladder paraganglioma does not always parallel the degree of catecholamine secretion, and absence of typical symptoms should not be equated with biochemical nonfunctionality. Our patient had no micturition-related headache, palpitations, sweating, or paroxysmal hypertension, yet plasma free NMN was approximately 1.35 × ULN, while MN, 3-MT, and 24-h urinary catecholamines were within their reference ranges. Guidelines recommend plasma free or urinary fractionated metanephrines as the principal biochemical tests for PPGL and emphasize the effects of posture, physiologic stress, medication, and analytical method on borderline results (13–16). In this report, biochemical activity was defined by a catecholamine or metanephrine value above the assay-specific upper reference limit, consistent with criteria used in published bladder paraganglioma series (20); the degree of secretion was interpreted in light of both the magnitude and reproducibility of the elevation. In this case, plasma samples were obtained after fasting and 20–30 min of supine rest and analyzed by LC-MS/MS. Because the mild NMN elevation was not repeated preoperatively, we classified the tumor as clinically silent but mildly biochemically functional, while avoiding interpretation of this isolated finding as evidence of marked catecholamine excess.

Imaging also contributed to the preoperative differential diagnosis. Both lesions were well circumscribed and hypervascular, with pronounced arterial enhancement of the larger lesion and further enhancement in the venous phase; cystoscopy showed intact, smooth mucosa with conspicuous surface vessels. This combination should raise suspicion for bladder paraganglioma. Compared with typical urothelial carcinoma, bladder paraganglioma more often shows submucosal or intramural growth, preserved mucosa, and strong enhancement, although leiomyoma, hemangioma, and other neuroendocrine tumors may show overlapping features (7, 11, 12). On histology, the Zellballen architecture, neuroendocrine marker expression, and lack of broad-spectrum cytokeratin staining further support the distinction. GATA3 expression may also be seen in paraganglioma and should not be interpreted in isolation as evidence of urothelial differentiation (26). MRI can provide additional tissue characterization and information on bladder-wall layers; in this patient it was not obtained because CT and cystoscopy were considered sufficient for lesion localization and operative planning.

Preoperative recognition of a potentially secretory bladder paraganglioma is important for perioperative management. Severe hypertension and arrhythmia have been reported during transurethral manipulation of an unrecognized lesion (17). After endocrinology consultation, our patient did not receive preoperative alpha-adrenergic blockade or a PPGL-specific volume-expansion regimen because she had no typical secretory symptoms and only mild biochemical abnormality. Continuous invasive arterial pressure, heart rate, and EtCO₂ monitoring were used during anesthesia, with treatment guided by hemodynamic changes; no sustained severe hemodynamic crisis occurred. This individual course should not be taken to mean that alpha-blockade or volume optimization can be routinely omitted. Patients with more substantial biochemical elevation, typical symptoms, or strong clinical suspicion of a functional PPGL should undergo guideline-based, multidisciplinary preoperative preparation.

Complete surgical excision remains the main treatment for bladder paraganglioma. Available approaches include TURBT, open partial cystectomy, laparoscopic or robot-assisted partial cystectomy, and several forms of pneumovesicoscopic resection (2, 18–25). TURBT is less invasive and may be suitable for selected small, predominantly intraluminal lesions, but piecemeal resection can compromise specimen integrity and assessment of the deep margin. Partial cystectomy more readily provides a full-thickness specimen but is more invasive, while laparoscopic and robotic approaches combine magnified visualization with bladder reconstruction. Pneumovesicoscopic resection creates an intravesical working space in which dissection, hemostasis, and bladder closure can be performed under direct vision. Table 3 summarizes the main technical features and current evidence for these approaches.

**Table 3:** Technical characteristics of common surgical approaches for bladder paraganglioma based on published case series and technical reports (2, 18–25).

Approach	Invasiveness	Specimen integrity	Operative view	Secretory risk	Bladder preservation	Evidence
TURBT	Low	May fragment; *en bloc* improves integrity	Endoluminal; deep margin may be limited	Present; manipulation may trigger release	Yes	Case series/retrospective
Open partial cystectomy	High	Usually intact	Direct exposure; full-thickness resection	Present	Yes	Case series/retrospective
Laparoscopic/robotic partial cystectomy	Moderate	Usually intact	Magnified view; full-thickness resection and closure	Present	Yes	Small series/case reports
Pneumovesicoscopic *en bloc* resection	Low to moderate	Intact specimen	Magnified intravesical view	Present; not eliminated	Yes	Case/technical reports

Pneumovesicum provides an intravesical CO₂ working space for laparoscopic instruments. Technical reports suggest that a gaseous medium may lessen the immediate visual obstruction caused by small amounts of blood or tissue debris and provide a relatively stable operative field; ultrasonic energy devices combine cutting and coagulation and may, in theory, reduce electrical stimulation associated with conventional monopolar resection (22–25). In this patient, both lesions were retrieved intact, estimated blood loss was approximately 10 mL, and no obvious obturator reflex or bladder perforation occurred. These observations support technical feasibility in this case but do not establish superiority over other surgical approaches.

Pneumovesicoscopic surgery also requires careful control of intravesical pressure and attention to CO₂ absorption. Published clinical series have used a range of pressures, commonly about 8–15 mmHg, with 10–12 mmHg frequently reported (22, 25). We used a STORZ constant-pressure system set at 11 mmHg for 106 min; the Foley catheter balloon was used to occlude the bladder outlet and reduce CO₂ leakage, and intraoperative EtCO₂ remained approximately 27–40 mmHg, with no clinically apparent CO₂-related complication. Pressure should be individualized to the patient, bladder capacity, and operative requirements, using the lowest pressure that provides a stable working space. From a technical standpoint, pneumovesicoscopic *en bloc* resection may be an option for selected localized lesions near the bladder neck, trigone, or ureteral orifices when precise intravesical dissection and an intact specimen are desirable. In our case, both ureteral orifices remained clearly visible and neither lesion involved the ureteral openings; therefore, prophylactic ureteral stents were not placed. However, the evidence remains limited to case reports and small technical series, and pneumovesicum does not eliminate catecholamine release caused by traction, cutting, or compression.

Histopathologically, bladder paraganglioma typically shows nested or organoid growth with a characteristic Zellballen architecture and neuroendocrine differentiation (21, 27). In our patient, the tumors were confined to the submucosa, with rare mitotic figures, no necrosis, no lymphovascular or perineural invasion, a Ki-67 index <1%, and retained SDHB expression. The intraoperative description of grossly complete *en bloc* removal refers to the surgical appearance, whereas tumor-free margins were established on histopathologic examination. Previous studies have associated necrosis, vascular invasion, increased mitotic activity, higher Ki-67 indices, and loss of SDHB expression with more aggressive behavior (21, 28, 29), but no single morphologic or immunohistochemical feature reliably predicts the long-term behavior of PPGL. Clinical course and continued surveillance remain essential.

Retained SDHB staining does not exclude all hereditary forms of PPGL. Because this patient had multifocal bladder tumors, hereditary susceptibility remains clinically relevant despite the negative family history. Genetic counseling and PPGL-related germline testing were recommended during follow-up but had not yet been performed at the time of manuscript revision (3).

Preoperative abdominal and pelvic CT showed normal adrenal glands and no additional paraganglioma-like lesion or definite metastatic disease within the imaged field. No whole-body functional imaging was performed, so disease outside the scanned field cannot be completely excluded. Given the multifocal bladder involvement, further whole-body anatomic or functional imaging may be considered during follow-up according to biochemical findings, genetic risk, and the clinical course.

At 12 months, cystoscopy showed no recurrence, indicating satisfactory short-term local control, but bladder paraganglioma can recur late or be followed by a new PPGL or metastatic disease. Long-term follow-up after PPGL resection is recommended (30). Plasma NMN and other metanephrines have not yet been repeated postoperatively in this patient. Our planned surveillance includes annual clinical assessment, annual plasma or urinary metanephrine testing, and annual cystoscopy, with cross-sectional or functional imaging added when indicated by biochemical findings, genetic risk, symptoms, or the clinical course. The single-case design, 12-month follow-up, lack of repeat testing for the mild preoperative NMN elevation, absence of completed germline testing and whole-body functional imaging, and the inability to compare this technique directly with other surgical approaches should be considered when interpreting the outcome.

## Conclusions

In summary, bladder paraganglioma should be considered when a hypervascular submucosal bladder mass has intact overlying mucosa, even in the absence of typical catecholamine-related symptoms. In this patient, pneumovesicoscopic *en bloc* resection allowed intact specimen retrieval and bladder-wall repair with an uncomplicated perioperative course. However, this is a single case with only 12 months of follow-up, and no conclusions can be drawn regarding comparative effectiveness or long-term oncologic safety. Optimal patient selection, long-term recurrence outcomes, and the broader safety profile of pneumovesicoscopic *en bloc* resection remain to be established in larger series with longer follow-up.

## Data Availability

The original contributions presented in the study are included in the article/Supplementary Material, further inquiries can be directed to the corresponding author.
